# Analysis of Longitudinal Stent Deformation by 3DStent Technology: An In Vitro Pilot Study

**DOI:** 10.1016/j.jscai.2025.103660

**Published:** 2025-06-10

**Authors:** Nicolas Amabile, Benjamin Honton, Damien Picart, Elsie Salvaudon, Liliane Ramus, Hakim Benamer

**Affiliations:** a Cardiology Department, Institut Cardiovasculaire Paris Sud, Massy, France; b Cardiology Department, Clinique Pasteur Toulouse, Toulouse, France; c GE HealthCare Research Center, Buc, France

**Keywords:** complication, coronary angiography, stent enhancement, stents

## Abstract

**Background:**

Longitudinal stent deformation (LSD) is a classical pitfall of aorto-ostial percutaneous coronary intervention that carries out a higher risk for adverse outcomes when not corrected. This in vitro pilot study tested the performances of the new 3DStent (3DS) imaging mode (GE HealthCare) for the diagnosis of LSD.

**Methods:**

A total of 41 coronary stents (diameter: 3.0 mm; length: 21-32 mm) were implanted in silicone tubes. LSD was created in 21 samples by crushing the proximal stent edge with a guiding catheter tip, whereas the others were left undeformed. All samples were analyzed by angiography methods including 3DS. The data were analyzed twice by 3 independent reviewers blinded to the characteristics of the samples. The diagnosis was based on the assessment of the following: (1) increased radio-opacity in the proximal stent section; (2) loss of stent borders parallelism; (3) loss of stent cells symmetry in its proximal part; (4) decreased stent length; and (5) decreased internal area in proximal stent section.

**Results:**

A total of 246 reconstructions were analyzed. The 3DS overall performances for LSD diagnosis were good: the global sensitivity and specificity were respectively 98.9% and 100% (positive predictive value: 100%; negative predictive value: 99%). The interobserver and intraobserver variabilities (Cohen’s k) ranged from k = 0.95 to k = 1.0 for the LSD diagnosis. The different individual item sensitivities ranged between 92% and 98% and their specificities between 99% and 100%.

**Conclusions:**

The 3DS imaging technology appeared to be efficient in identifying LSD in vitro. Whether these results could be translated in vivo needs to be assessed.

## Introduction

Left main and aorto-ostial percutaneous coronary interventions (PCI) are still considered as complex procedures and remain a challenge in daily practice.^1^ These interventions are technically demanding and could also lead to incomplete results or mechanical complications, including longitudinal stent deformation (LSD).^1^ LSD is related to stent shortening in the longitudinal axis following initially successful device deployment. This complication induces device structure folding (“accordion” effect), and struts malapposition and stent underexpansion.^2^ The most frequent reasons for LSD are collisions between the guiding catheter tip and the proximal edge of the platform (especially when withdrawing equipment back through a stent).^2^^,^^3^ When not corrected, this abnormality could promote complications such as stent thrombosis or intrastent restenosis and then lead to a higher incidence of major adverse clinical events.^4^, ^5^, ^6^

The diagnosis of LSD is challenging and most frequently relies on conventional angiography. However, recent analyses showed that stent deformation was underestimated by angiography alone and could be present in up to 9.3% of bifurcation and 18.5% of left main PCI.^4^ Intracoronary imaging is useful for LSD identification but the associated signs (either by optical coherence tomography [OCT] or intravascular ultrasound) are discrete and these tools might present some limitations.^5^^,^^7^^,^^8^

3DStent (3DS; GE HealthCare) is a new angiography-based technology that allows visualization of stents in 3D during the procedure. It is based on the C-arm motion compensated computed tomography principle and improves the analysis of the stent structure and dimensions compared to conventional 2D stent-enhancement tools.^9^ Preliminary reports showed promising results for stent expansion assessment,^9^, ^10^, ^11^ but the technique was never evaluated for the detection of deformed stent structure. The aim of this pilot in vitro work was to investigate the feasibility and accuracy of 3DS technology for the diagnosis of LSD.

## Materials and methods

### LSD in vitro model

**Figure 1:**
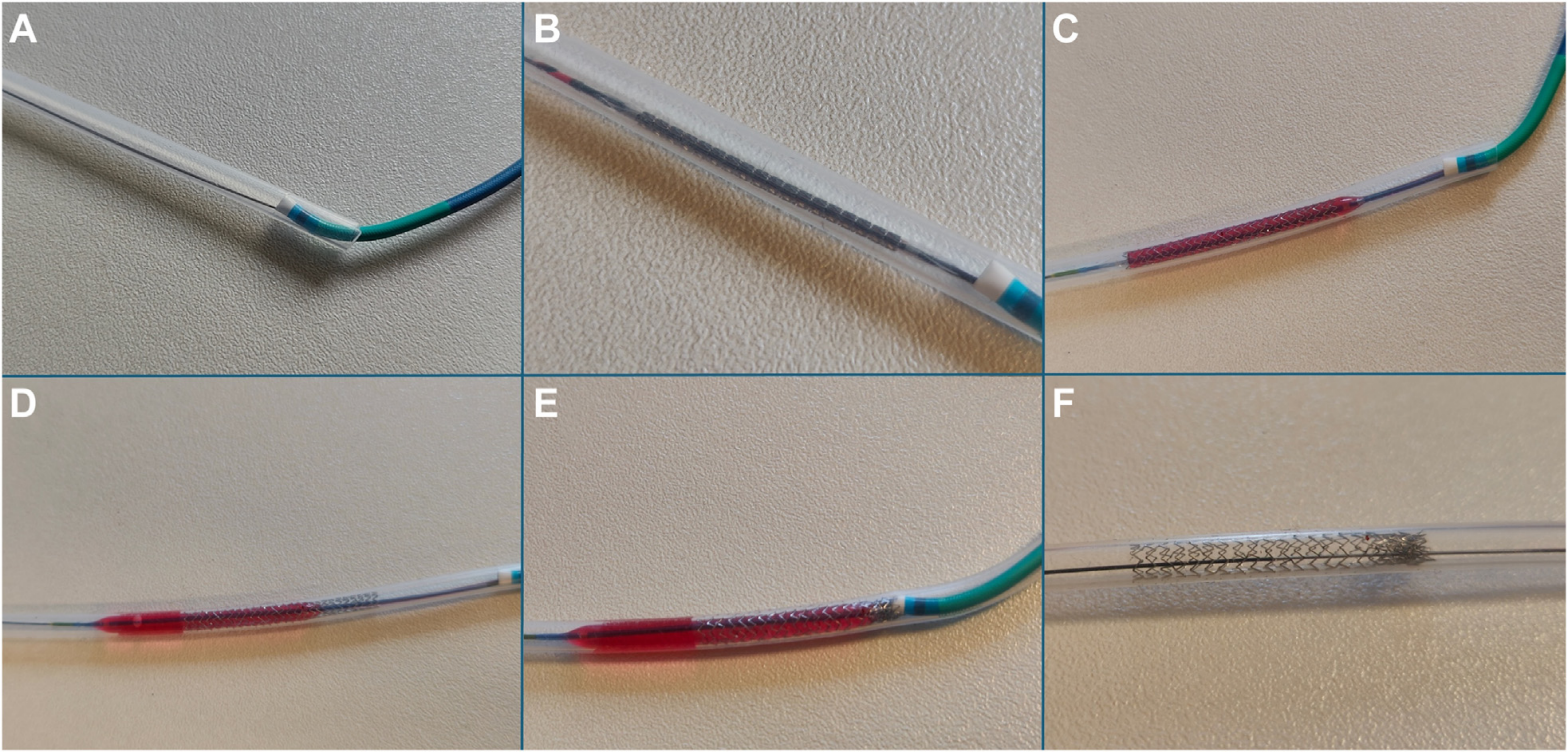
**In vitro stent implantation and longitudinal deformation maneuvers.** The silicon tube is canulated by the guiding catheter and a 0.014-inch wire is placed (A). The 3.0 mm diameter stent is placed in the target zone (B) and the balloon is inflated below the nominal expansion pressure (C). The balloon is then deflated, placed more distal, and reinflated at nominal pressure for 30 seconds (D). During the second inflation, the guiding catheter is advanced and collides with the proximal stent edge (E). The balloon is deflated and retrieved from the tube leaving the deformed stent on site (F).

We implanted 3.0 mm diameter coronary stents (TAXUS, Boston Scientific and ULTIMASTER, Terumo; length: 21 to 32 mm) in silicone tubes and included the samples in 2 separate groups. In the first group (LSD group), a dedicated compression sequence was applied (Figure 1). Briefly, a 6F Judkins right guiding catheter was inserted in the tube’s proximal orifice. The tube was wired with a 0.014-inch dedicated wire (Runthrough wire, Terumo), and the stent was initially placed 10 mm distal to the proximal edge of the tube and inflated under the device's nominal pressure. The balloon was then deflated and advanced more distal (30% to 50% of the total stent length) and reinflated up to the nominal pressure to get full expansion of the distal section of the device. During the second inflation, the guiding catheter was advanced into the tube to collide with the stent proximal section and create longitudinal compression. This collision maneuver was repeated 3 times. In the second group (control group), the stent was placed 10 mm distal to the proximal edge of the tube, through a 6F Judkins right guiding catheter and inflated to the device nominal pressure without applying the LSD sequence. After each stent implantation in both groups, the balloon was deflated and its shaft was cut and fixed to the tube to prevent balloon retrieval within the stent. Each sample was then covered with an opaque adhesive tape and labeled for future analyses.

### 3DS acquisition and reviews

The anonymized samples (stents + deflated balloons in place) were placed on a moving phantom surface and analyzed with the Allia IGS 5 Pulse angiography system (GE HealthCare). The 3DS acquisition process has been previously described elsewhere.^11^ The first step of the procedure was to center the samples within the field of view by adjusting the table height and position in frontal and lateral views. A “test spin” phase (with no x-ray exposure) was then performed by automated rotation at low speed to ensure the absence of collisions with the equipment present in the room. The rotational acquisition was finally performed during an automatic C-arm rotation that covered 200° from RAO 100° to LAO 100°.

The rotational angiography images were processed through a dedicated C-arm motion compensated computed tomography software. This approach is derived from the cone beam computed tomography technology but involves in addition a compensation of the heart’s anatomic motions. These movements are determined by tracking deflated balloon markers as preidentified landmarks in the different projections to precisely determine the position of the stent in the space. The reconstruction algorithm produces 0.1 mm voxel-based images that can be visualized with different standard rendering methods. The 3D reconstructed stent can be reviewed in volume rendering or maximum intensity projection views (Supplemental Videos 1 and 2). The stent can also be analyzed in a multiplanar reformat to get a representation of the device in 3 perpendicular views defined according to the axis defined by the 2 markers: an axial view perpendicular to this axis, showing the cross-sections of the stent, and 2 longitudinal views with this axis included in the slice. Measurements are possible in these different views allowing for the obtention of the stent diameters and areas at any position or orientation in the axial view. The views can be adjusted to make sure that the measurements are placed in cross-sections perpendicular to the stent axis. Stent diameter and area can be measured at different levels of the stent, and the total length of the stent can be measured as well.

The 3DS data were analyzed twice on a dedicated offline review station in random order by 3 reviewers blinded to the sample group.

**Figure 2:**
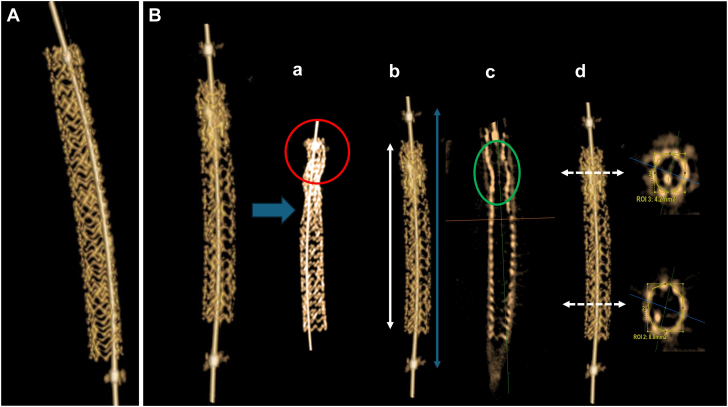
**Examples of nondeformed (A) and deformed stents (B) analyzed by 3DStent imaging.** The diagnosis of longitudinal stent deformation was based on the presence of increased local radio-opacity and loss of stent cells symmetry (a), decreased stent length (white arrow) compared to theoretical length (based on distance between balloon markers or blue arrow or (b), presence of struts indentation and loss of stent borders parallelism (c) and decreased proximal stent area compared to distal stent area (d).

The reviewers had to assess the presence of the following elements which were previously identified in our initial experience (Figure 2): (1) increased radio-opacity in proximal stent section; (2) loss of stent border parallelism and presence of struts indentation; (3) decreased stent length (>0.7 mm decrease in stent length compared to expected or theoretical stent length); (4) loss of stent cell symmetry in proximal part; (5) decreased internal stent area (more than 15% decrease of proximal stent internal area compared to its distal part area). The presence of any of these signs was assessed and graded to 0 or 1, leading to the calculation of a compression score ranging from 0 to 5 as the sum of the individualized items. Finally, each reviewer had to conclude the presence or not of LSD based on this analysis.

### End points

The coprimary end points of the study were the sensitivity and specificity of the 3DS tool for the adequate overall diagnosis of LSD among samples. In addition, we also assessed the sensitivity and specificity of the different individualized compression score items (see before) as well as the interobserver and intraobserver variability for overall diagnosis and individual signs assessment.

### Statistical analysis

Statistical analysis was performed with SPSS 28.0 software (IBM Corp) and GraphPad Prism 10.0 (GraphPad). Qualitative data were expressed in percent and continuous data were expressed in median (IQR). The differences between the variables were compared by the χ^2^ or Fisher test for qualitative variables and by the Mann-Whitney *U* or Student's t test for quantitative variables. The intraobserver and interobserver variabilities for LSD diagnosis and individual compression signs were calculated according to Cohen’s k method. The correlation between quantitative variables measurements was evaluated with the Pearson correlations coefficient and the Bland-Altman plot method. Receiver operating characteristic analysis was performed to assess the discriminative power of the LSD score for stent compression identification. All tests were 2-sided with an alpha level of 5%.

## Results

A total of 41 stents were implanted in the silicone tubes, including 21 in the LSD group and 20 in the control group. The median stent lengths before implantation were 32 (IQR: 27; 32) mm in the control and 32 (IQR 28; 32) mm in the LSD groups (*P* = 1.0). 3DS image acquisition was performed in all samples, which were analyzed twice by the 3 reviewers. Hence, a total of 246 3DS images were reviewed. In 1 sample from the control group, the stent length measurement was not feasible because of technical issues.

### 3DS diagnostic performances

The stent lengths and minimal areas values were significantly higher in the control group compared to the LSD group (32.0 [24-32] vs 29.3 [23.3-31.0] mm and 7.2 [6.5-7.6] vs 5.0 [4.3-5.6] mm^2^, respectively; *P* < .001 for both). Tables 1 and 2 present the performances of 3DS analysis for the overall diagnosis of LSD and individual items assessment. The tool was accurate, as shown by the 99.2% sensitivity and 100% specificity for LSD identification. In addition, sensitivity ranged from 89.7% to 97.6% and specificity from 96.5% to 100% for individual LSD item identification.

**Table 1:** 3DStent overall performances for longitudinal stent deformation diagnosis.

Parameters	Rev #1 initial	Rev #1 redo	Rev #2 initial	Rev #2 redo	Rev #3 initial	Rev #3 redo	Total
Individual analysis							
True positive, n	20	21	21	21	21	21	125
False positive, n	0	0	0	0	0	0	0
True negative, n	20	20	20	20	20	20	120
False negative, n	1	0	0	0	0	0	1
Performance							
Sensitivity, %	95.2	100	100	100	100	100	99.2
Specificity, %	100	100	100	100	100	100	100
PPV, %	100	100	100	100	100	100	100
NPV, %	95.2	100	100	100	100	100	99.2

NPV, negative predictive value; PPV, positive predictive value; Rev, reviewer.

**Table 2:** 3DStent performance for individual items assessment.

Parameters	Increased radio-opacity	Stent indentation	Asymmetrical stent cells	Decreased stent length	Decreased internal stent area
Individual analysis					
True positive, n	123	118	119	113	123
False positive, n	0	0	0	4	1
True negative, n	120	120	120	110	119
False negative, n	3	8	7	13	3
Performance					
Sensitivity, %	97.6	93.7	94.4	89.7	97.6
Specificity, %	100.0	100.0	100.0	96.5	99.2
PPV, %	100.0	100.0	100.0	96.6	99.2
NPV, %	97.6	93.8	94.5	89.4	97.5

NPV, negative predictive value; PPV, positive predictive value.

### Reproducibility and variability

Table 3 reports the intraobserver and interobserver variabilities for the different analyses. The Cohen’s k ranged from 0.95 to 1.0 for global LSD diagnosis and from 0.8 to 1.0 for the dedicated individual items analysis. These results suggested excellent intraobserver and interobserver reproducibility.

**Table 3:** Intraobserver and interobserver variabilities for global longitudinal stent deformation diagnosis and individual items assessment.

Parameters	Global diagnosis	Increased radio-opacity	Stent indentation	Asymmetrical stent cells	Decreased stent length	Decreased internal stent area
Intraobserver variability						
Rev #1	0.95	0.9	0.95	0.95	0.9	0.95
Rev #2	1.0	0.9	0.80	0.80	0.95	0.90
Rev #3	1.0	1.0	1.0	0.90	1.0	0.95
Interobserver variability						
Rev #1 vs Rev #2	0.98	0.95	0.93	0.93	0.90	0.98
Rev #1 vs Rev #3	0.98	0.98	0.87	0.88	0.88	1.0
Rev #2 vs Rev #3	1.0	0.95	0.88	0.90	0.98	0.98

Rev, reviewer.

**Figure 3:**
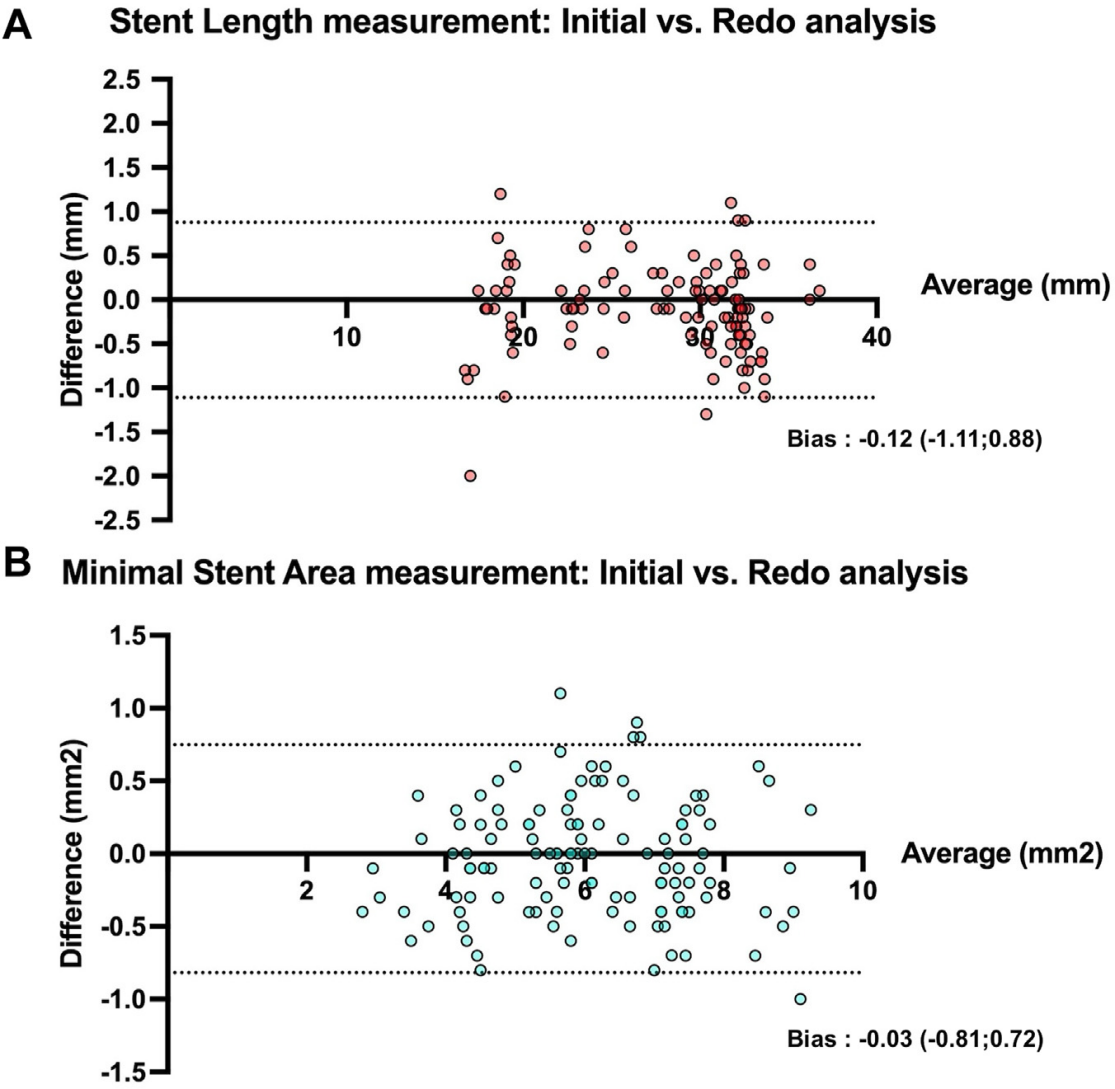
**Bland-Altman plots analyses for stent length (A) and minimal area (B) in initial vs redo analyses**.

Figure 3 shows the results of the Bland-Altman plots for stent length and minimal stent area (MSA) between initial and redo measurements. In these analyses, the overall bias was minimal, yet we observed a nonsignificant trend toward smaller bias in the case of longer and larger devices: –0.17 ± 0.47 vs –0.05 ± 0.54 (*P* = .1, *t* test) for stent length ≥30 mm vs <30 mm, respectively and 0.01 ± 0.45 vs –0.09 ± 0.32 (*P* = .2, *t* test) for MSA ≥6 mm^2^ vs MSA <6 mm^2^, respectively.

In addition, the stent lengths and minimal areas were highly correlated between the 2 series of measurements: Pearson’s r^2^ was equal to 0.99 (*P* < .001) for both variables. Finally, we also calculated the r^2^ correlation coefficients and bias according to the Bland-Altman methods for intraobserver and interobserver comparison. These data are presented in Table 4.

**Table 4:** Intraobserver and interobserver correlations and variabilities for stent length and MSA values assessment by 3DS imaging.

Parameter	Rev #1 initial vs redo	Rev #2 initial vs redo	Rev #3 initial vs redo	Rev #1 vs Rev #2	Rev #1 vs Rev #3	Rev #2 vs Rev #3
Stent length						
Pearson’s R^2^	0.99	0.99	0.99	0.98	0.99	0.99
*P* value	.0001	.0001	.0001	.0001	.0001	.0001
Bland-Altman bias	–0.05	–0.17	–0.11	–0.30	–0.29	0.01
95% CI	–0.98 to 0.88	–1.25 to 0.90	–1.01 to 0.80	–2.01 to 1.41	–1.60 to 1.02	–1.32; 1.34
MSA						
Pearson’s R^2^	0.94	0.90	0.94	0.79	0.79	0.83
*P* value	.0001	.0001	.0001	.0001	.0001	.0001
Bland-Altman bias	–0.16	0.12	–0.05	1.66	0.51	–1.14
95% CI	–0.91 to 0.57	–0.75 to 1.0	–0.67 to 0.56	0.32 to 2.99	–0.82 to 1.85	–2.13 to –0.16

3DS, 3DStent; CI, confidence interval; MSA, minimal stent area; Rev, reviewer.

### Compression score

The median compression score was 5.0 (4.0-5.0) and 0 (0-0) in the LSD and control groups, respectively (*P* < .001). The receiver operating characteristic curve analysis showed that compression score >1 was the optimal cut-off value to predict actual LSD (area under the curve, 1.0; 95% CI, 1.0-1.0; *P* < .001).

## Discussion

The current study analyzed the potential application of the 3DS imaging technology to identify the presence of LSD in an in vitro mode, according to prespecified criteria. Our results suggest that this strategy was feasible, accurate, and reproducible.

LSD is a classical complication of PCI in which the coronary stent is shortened or elongated along its longitudinal axis. LSD is mostly related to the collision between the guiding catheter tip (or guiding catheter extension) and the proximal edge of the stent, which can be favored by withdrawing equipment back through the device. Thus, LSD is most frequently observed during the proximal or ostial right coronary artery of the left main stem PCI. In addition, LSD can also be observed in the case of deep guiding catheter extension intubation in tortuous and/or calcified vessels and involves smaller stents in a more distal position.^12^ LSD heavily affects the device integrity by folding its structure and creating stent underexpansion, struts malapposition and overlap, and edge dissection. When not corrected, these mechanical abnormalities could trigger potential complications such as stent thrombosis, restenosis, and eventually target lesion revascularization.^4^^,^^6^^,^^13^^,^^14^ However, the real incidence of LSD is probably underestimated: yet some conventional angiography-based analyses evaluated its frequency up to 1.2%^2^^,^^3^^,^^6^; the most recent analyses based on intracoronary imaging reported that LSD might be present in 6.5% to 19.5% of left main stem PCI.^4^^,^^13^

The diagnosis of LSD remains challenging as conventional coronary angiography is limited in this situation. The usual sign is the presence of a ring-like image caused by the overlapping stent struts associated with a shortened device length along its long axis.^15^ However, the sensitivity of the technique may vary, according to the penetration of the x-ray through different coronary stents (that also depends on their design and radio-opacity), the presence of an underlying balloon within the device, and the clinical experience of physicians.^3^^,^^16^ Stent-enhancement techniques could improve the diagnosis accuracy, but this requires different orthogonal views to fully assess the stent structure. Intracoronary imaging techniques (intravascular ultrasound or OCT) are currently considered the gold standard to identify LSD, usually depicting the association of localized struts overlapping segments (double or triple struts layers), struts malapposition, stent underexpansion, and potential edge dissection.^5^^,^^7^^,^^8^ However, these signs could be subtle, and their assessment is challenging for nonexperts. In addition, OCT remains difficult to perform in aorto-ostial lesions although this is a preferential location for LSD and requires contrast medium additional injection.^17^ Thus, there is still room for improvement in our diagnostic tools.

Our data show that 3DS was feasible and efficient for LSD detection. 3DS is a novel rotational, noninjected angiography-based imaging that features 3-dimensional and multiplanar stent reconstruction, allowing for device structure analysis and quantitative stent area and length assessment. Previous preliminary experiences reported adequate correlation with OCT measurements for MSA.^9^, ^10^, ^11^ However, this is the first time, to the best of our knowledge, that the tool was used for stent deformation analysis. Compared with conventional angiography and stent-enhancement techniques, 3DS offers several advantages: volumetric analysis of the device, the possibility of multiple longitudinal and transverse sections, and the ability to precisely measure stent dimensions.^11^ Using and combining preestablished criteria, 3DS showed excellent sensitivity and predictive positive value for LSD diagnosis in this model (Central Illustration). Of note, we observed excellent intraobserver and interobserver variabilities, even for the measurement of stent dimensions. Hence, the correlations between initial and redo measurements, as well as between reviewers were good for stent length and MSA assessment, yet we cannot exclude an influence of the baseline device dimensions on results as suggested by the nonsignificant differences observed in the Bland-Altman biases. However, the translation of our results to in vivo in patients remains to be evaluated. The application of the technique to human beings could be hampered by some limitations. The penetration of x-rays within the heart could be affected by some individual parameters, such as high body mass index. Moreover, the presence of underlying vascular calcifications around the stent might also interact with the strut signal and affect the image quality. These different points will have to be assessed in the future.

**Central Illustration:**
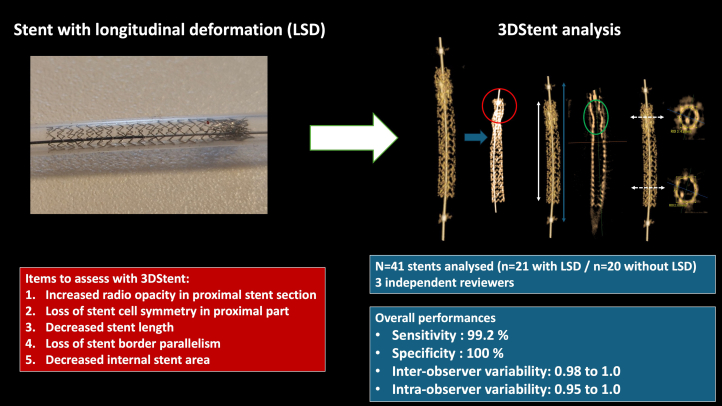
**Longitudinal stent deformation (LSD) assessment by 3DStenttechnology: Items to analyze and global diagnosis performances.**

### Limitations

Although our study presents the largest series of 3DS images for LSD reported so far, we acknowledge some limitations. This is an experimental in vitro study using stent implantation in silicone tubes and the findings observed might not be entirely translatable to in vivo arteries (see the earlier paragraph). In addition, the current analysis focused on longitudinal compression, and we did not study other types of stent deformation such as elongation, partial crush, severe struts malapposition, or consequences of abluminal wiring that are also observed in vivo.^4^ Finally, even though 3DS images are easily interpretable, it is difficult to know whether the results could have been improved with greater reviewer experience.

In conclusion, the present results of this in vitro pilot study suggest that 3DS technology could efficiently detect LSD. Whether these preliminary observations could be reproduced in vivo and this strategy be used in clinical practice needs to be assessed.

## Supplementary material

Supplemental Video 1 An example of stent with LSD analyzed with 3DS technology.

Supplemental Video 2 An example of intact stent analyzed with 3DS technology.
